# High-Conductivity and Moisture-Tolerant Sulfide Electrolyte Enabled by Bifunctional Coating for Practical All-Solid-State Lithium Batteries

**DOI:** 10.1007/s40820-026-02353-y

**Published:** 2026-10-05

**Authors:** Dabing Li, Hongjie Xu, Guoqin Cao, Fanfan Liu, Jinjin Ban, Shilin Zhang, Li-Zhen Fan, Junhua Hu

**Affiliations:** 1 School of Materials Science and Engineering, State Center for International Cooperation on Designer Low-carbon and Environmental Materials (CDLCEM), Zhengzhou University, Zhengzhou, 450001 People’s Republic of China; 2 School of Materials Science and Engineering, North China University of Water Resources and Electric Power, Zhengzhou, 450046 People’s Republic of China; 3 Institute of Advanced Materials and Technology, University of Science and Technology Beijing, Beijing, 100083 People’s Republic of China

**Keywords:** All-solid-state lithium batteries, Sulfide electrolytes, Surface modification, Moisture tolerance, Long cycling stability

## Abstract

The pursuit of safer and higher-energy-density batteries has driven the advancement of all-solid-state lithium batteries (ASSLBs). Among various material systems, sulfide solid-state electrolytes (SEs) stand out as highly promising candidates owing to their exceptional ionic conductivity. Nevertheless, their extreme sensitivity to moisture results in the release of toxic H_2_S and rapid structural degradation, which severely impedes their scalable manufacturing under ambient conditions. A bifunctional (3-mercaptopropyl)trimethoxysilane (MPTMS) coating strategy is herein presented to shield Li_5.5_PS_4.5_Cl_0.8_Br_0.7_ (LPSCBr) from moisture-induced degradation. The MPTMS layer forms a hydrophobic barrier of approximately 10 nm, which reduces H_2_S emission by 93.0% after 35 min of exposure to 30% relative humidity, while maintaining 83.1% of the initial ionic conductivity (10.8 mS cm^−1^). This approach tackles the core challenge associated with sulfide solid-state electrolytes: the realization of compatibility with controlled ambient air without sacrificing critical electrochemical properties. The modified electrolyte maintains a high ionic conductivity (> 5.7 mS cm^−1^) even after 10 h of air exposure. When applied in all-solid-state cells paired with LiNi_0.83_Co_0.11_Mn_0.06_O_2_ cathodes, it exhibits remarkable electrochemical performance, delivering a capacity retention of 84.6% after 1000 cycles. By enabling moisture-tolerant processing, MPTMS-modified LPSCBr SEs lay a solid foundation for the scalable, high-performance, and cost-effective production of ASSLBs.

**Supplementary Information:**

The online version contains supplementary material available at https://doi.org/10.1007/s40820-026-02353-y.

## Highlights

- (3-Mercaptopropyl)trimethoxysilane (MPTMS) forms a hydrophobic coating on Li_5.5_PS_4.5_Cl_0.8_Br_0.7_ (LPSCBr) via synergistic covalent bonding of dual functional groups (–SH and –Si(OCH_3_)_3_).
- The siloxane network blocks moisture, and S–S bonds stabilize the PS_4_^3−^ framework to inhibit electrolyte hydrolysis fundamentally.
- The bifunctional MPTMS coating on LPSCBr simultaneously enhances electrolyte moisture resistance and cathode interfacial stability.

**Supplementary Information:**

The online version contains supplementary material available at https://doi.org/10.1007/s40820-026-02353-y.

## Graphical abstract

**Figure:**
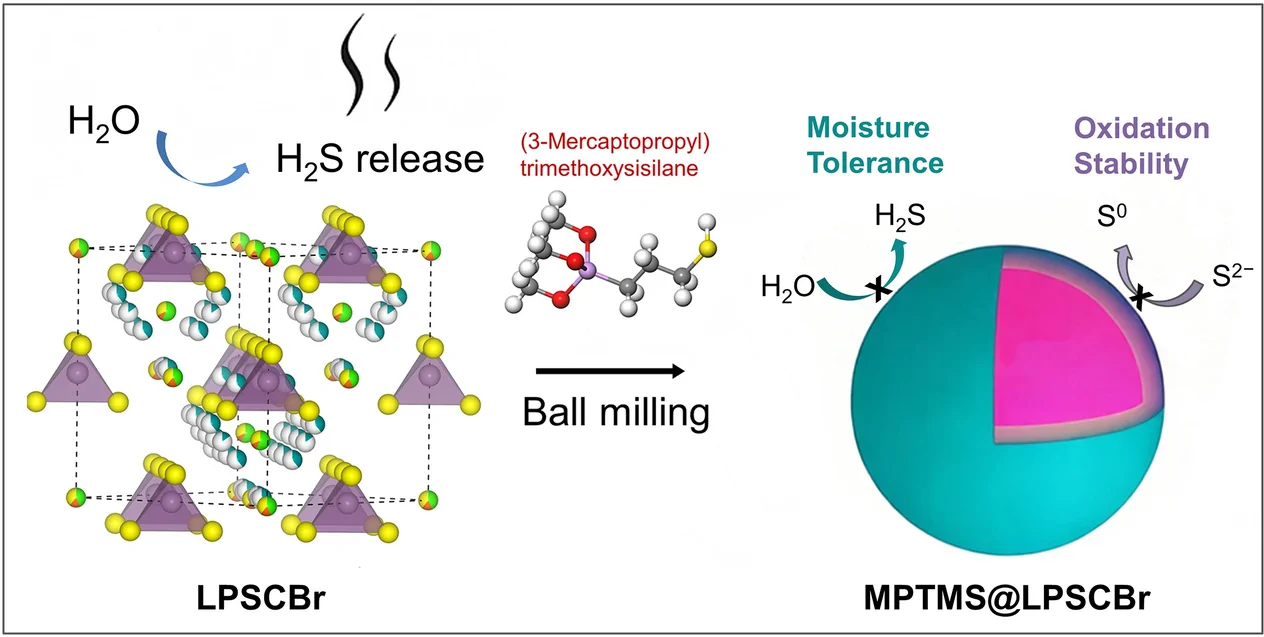

## Introduction

Lithium-ion batteries (LIBs) hold a dominant position in consumer electronics, electric vehicles, and grid energy storage applications, primarily due to their high energy density and excellent rechargeability [1]. In recent years, all-solid-state lithium batteries (ASSLBs) have emerged as a revolutionary technological advancement, which employ solid electrolyte (SE) technology to overcome the inherent limitations of liquid-electrolyte-based LIBs. ASSLBs eliminate the flammability risks associated with liquid electrolytes, enable stable operation over a wide temperature range, and support flexible device configurations. These advantages open up new possibilities for the application of high-voltage cathodes and lithium-metal anodes, thereby further boosting battery performance [2, 3]. Sulfide-based SEs have attracted significant attention due to their high lithium-ion conductivity, typically ranging from 10^−4^ to 10^−2^ S cm^−1^ at ambient temperature, along with favorable interfacial stability and mechanical flexibility [4–6]. However, their practical deployment faces critical challenges, especially poor air stability. These materials undergo irreversible reactions with moisture in the air, leading to the release of toxic hydrogen sulfide (H_2_S) and triggering structural degradation. Such degradation significantly reduces the ionic conductivity of the electrolytes and increases interfacial resistance [7], which not only complicates large-scale manufacturing under ambient conditions but also impairs the long-term cycling stability of batteries [8].

To solve this critical problem, researchers have developed a variety of modification strategies, among which composite preparation and anionic/cationic doping are the most widely explored. Hayashi et al. reported that composite sulfide electrolytes incorporating metal oxides (e.g., ZnO, Fe_2_O_3_, Bi_2_O_3_) can chemically adsorb H_2_S through exothermic reactions (ΔG < 0). However, the addition of metal oxides such as ZnO to Li_3_PS_4_ compromises the ionic conductivity of the electrolyte, as higher oxide contents lead to increased interfacial impedance [9]. In general, an ideal protection strategy should enhance the air stability without impairing the inherent ionic transport capability or redox stability of the electrolyte, while maintaining its high conductivity and good interfacial compatibility with electrodes. Based on the hard/soft acid–base (HSAB) theory [10], soft acids (e.g., As^5+^, Sb^5+^, Sn^4+^, Cu^2+^) tend to preferentially bind to soft bases (S^2−^), which can counteract the O^2−^-driven hydrolysis reaction of sulfide electrolytes [11–14]. For instance, Sb-doped Li_10_Ge(P_0.925_Sb_0.075_)_2_S_12_ retained 82% of its initial ionic conductivity after exposure to air, whereas the undoped Li_10_GeP_2_S_12_ suffered a 46% loss in ionic conductivity. The soft acidity of Sb ensures the formation of stronger covalent Sb–S bonds compared with those of P, thereby improving the air stability of the electrolyte [13]. Nevertheless, the stability advantage achieved by this substitution is partially offset by the reduced electrochemical reduction stability and increased material cost. Recently, the construction of an interfacial passivation layer on the surface of sulfide solid electrolytes has been proven to be an effective approach to enhance their air stability. These interfacial passivation layers include Lewis acid additives [15], inorganic coatings (e.g., LiF [16], Al_2_O_3_ [17]), and organic coatings (e.g., C_5_H_11_Br [18], 1H,1H,2H,2H-perfluorodecyltriethoxysilane [19], 1-undecanethiol [20], polydimethylsiloxane [21]). These coating layers mainly slow down the reaction between sulfide electrolytes and moisture in the air through physical isolation, chemical adsorption, or reaction inhibition mechanisms. Wu et al. prepared a fluoropolysiloxane protective layer on Li_1.4_Al_0.4_Ti_1.6_(PO_4_)_3_ nanoparticles via the hydrolysis and condensation reactions of tetraethyl orthosilicate (TEOS) and 1H,1H,2H,2H-perfluorodecyltriethoxysilane (PFDTES) molecules on the nanoparticle surface [19]. Benefiting from this protective layer, the modified Li_6_PS_5_Cl electrolyte could withstand extreme exposure conditions in high-humidity environments (relative humidity, RH = 70%). However, only a limited number of nonpolar solvents have been identified that do not chemically interact with the Li_6_PS_5_Cl surface [22]. The chemical degradation of sulfide SEs in some solvents can be attributed to the strong Lewis basicity of highly polar solvents [23]. Specifically, polar solvents contain electronegative atoms bearing lone electron pairs such as nitrogen and oxygen, which readily coordinate with hard acid sites like pentavalent phosphorus within sulfide solid electrolytes and trigger electrolyte chemical degradation. Furthermore, the stability of the interface between sulfide solid electrolytes and oxide cathodes is crucial for the development of ASSLBs with high safety and long cycling stability. Multiple obstacles exist at such interfaces, covering physical drawbacks including electrode volume fluctuation and poor interfacial adhesion, alongside chemical side reactions such as solid electrolyte breakdown, space-charge layer generation, and cross-element interdiffusion [24].

(3-Mercaptopropyl)trimethoxysilane (MPTMS) is ideal for coating: Dual functional groups (–SH and –Si–OCH_3_) enable fast, stable bonding [25], high sulfide compatibility without pre-treatments [26], and cost-efficiency for large-scale use. In this work, we introduce bifunctional MPTMS, which contains both thiol (–SH) and methoxysilane (–Si(OCH_3_)_3_) groups, onto the surface of the sulfide electrolyte Li_5.5_PS_4.5_Cl_0.8_Br_0.7_ (LPSCBr) via a mechanical ball-milling method. When exposed to 30% relative humidity at 25 °C, the MPTMS-modified LPSCBr (MPTMS@LPSCBr) effectively suppresses H_2_S release by 93.0% and retains 83.1% of its initial ionic conductivity (10.8 mS cm^−1^), which is attributed to the ultrathin and highly compatible MPTMS coating. Furthermore, we systematically investigate the interfacial bonding mechanism, moisture tolerance, and electrochemical performance of the modified electrolyte. When applied in all-solid-state batteries with LiNi_0.83_Co_0.11_Mn_0.06_O_2_ (NCM83) cathodes, the modified electrolyte exhibits excellent air stability and cycling performance, achieving a capacity retention of 84.6% after 1000 cycles. The MPTMS coating on LPSCBr also elevates the oxidation tolerance and interfacial matching between electrolyte and cathode. Additionally, the all-solid-state pouch cell configured with NCM83-MPTMS@LPSCBr composite cathode, LPSCBr film electrolyte, and μ-Si anode demonstrates stable cycling performance. These breakthroughs represent a critical step forward in advancing sulfide solid-state electrolytes from laboratory research to industrial application, laying a solid technical foundation for the development of air-stable, high-energy-density all-solid-state battery systems.

## Results and Discussion

### Surface Modification of LPSCBr Sulfide Electrolyte via MPTMS Coating

Sulfide solid electrolytes such as argyrodite-type LPSCBr are highly promising for ASSLBs, owing to their high lithium-ion conductivity and favorable mechanical deformability. However, their intrinsic moisture sensitivity severely hinders ambient air processing, as hydrolysis generates toxic H_2_S and causes structural degradation and ionic conductivity decay. Herein, a conformal MPTMS coating layer was grafted onto LPSCBr via a mechanochemical ball-milling strategy (10:1 weight ratio of LPSCBr to MPTMS). The dual functional groups of MPTMS work synergistically for moisture protection: The methoxysilane groups form a hydrophobic siloxane network, while the thiol groups form S–S bonds with surface sulfur species of LPSCBr, collectively enhancing the moisture stability.

1 $${\mathrm{HS}} - \left( {{\mathrm{CH}}_{2} } \right)_{3} {-}{\mathrm{Si}}\left( {{\mathrm{OCH}}_{3} } \right)_{3} + 3{\mathrm{H}}_{2} {\mathrm{O}} \to ^{{{\mathrm{RT}}}} {\text{ HS}} - \left( {{\mathrm{CH}}_{2} } \right)_{3} - {\mathrm{Si}}\left( {{\mathrm{OH}}} \right)_{3} + 3{\mathrm{CH}}_{3} {\mathrm{OH}}$$

2 $${\mathrm{HS}} - \left( {{\mathrm{CH}}_{2} } \right)_{3} - {\mathrm{Si}}\left( {{\mathrm{OH}}} \right)_{3} + {\mathrm{HO}} - {\mathrm{S}} \to ^{{{\mathrm{RT}}}} {\mathrm{HS}} - \left( {{\mathrm{CH}}_{2} } \right)_{3} - {\mathrm{Si}}\left( {{\mathrm{OH}}} \right)_{2} - {\mathrm{O}} - {\mathrm{S}} + {\mathrm{H}}_{2} {\mathrm{O}}$$

3 $$n{\mathrm{HS}} - \left( {{\mathrm{CH}}_{2} } \right)_{3} - {\mathrm{Si}}\left( {{\mathrm{OH}}} \right)_{2} - {\mathrm{O}} \to ^{{{\mathrm{RT}}}} \left[ {{\mathrm{Si}} - {\mathrm{O}}{-}{\mathrm{Si}}^{ - } } \right]_{n} {\text{cross - linked network}}$$

Upon exposure to trace moisture, hydrolysis of –Si(OCH_3_)_3_ generates reactive silanol groups, which condense with –OH species on the LPSCBr surface. This yields a cross-linked siloxane framework covalently anchored to the electrolyte, effectively suppressing parasitic reactions with atmospheric H_2_O while maintaining high ionic conductivity. Concurrently, the thiol group facilitates the formation of covalent S–S bonds with surface sulfur species in LPSCBr. The structural and chemical properties of MPTMS-coated LPSCBr were characterized through X-ray diffraction (XRD), Raman spectroscopy, solid-state nuclear magnetic resonance (NMR), scanning electron microscopy (SEM), high-resolution transmission electron microscopy (HRTEM), Fourier-transform infrared (FTIR) spectroscopy, and X-ray photoelectron spectroscopy (XPS).

**Fig. 1:**
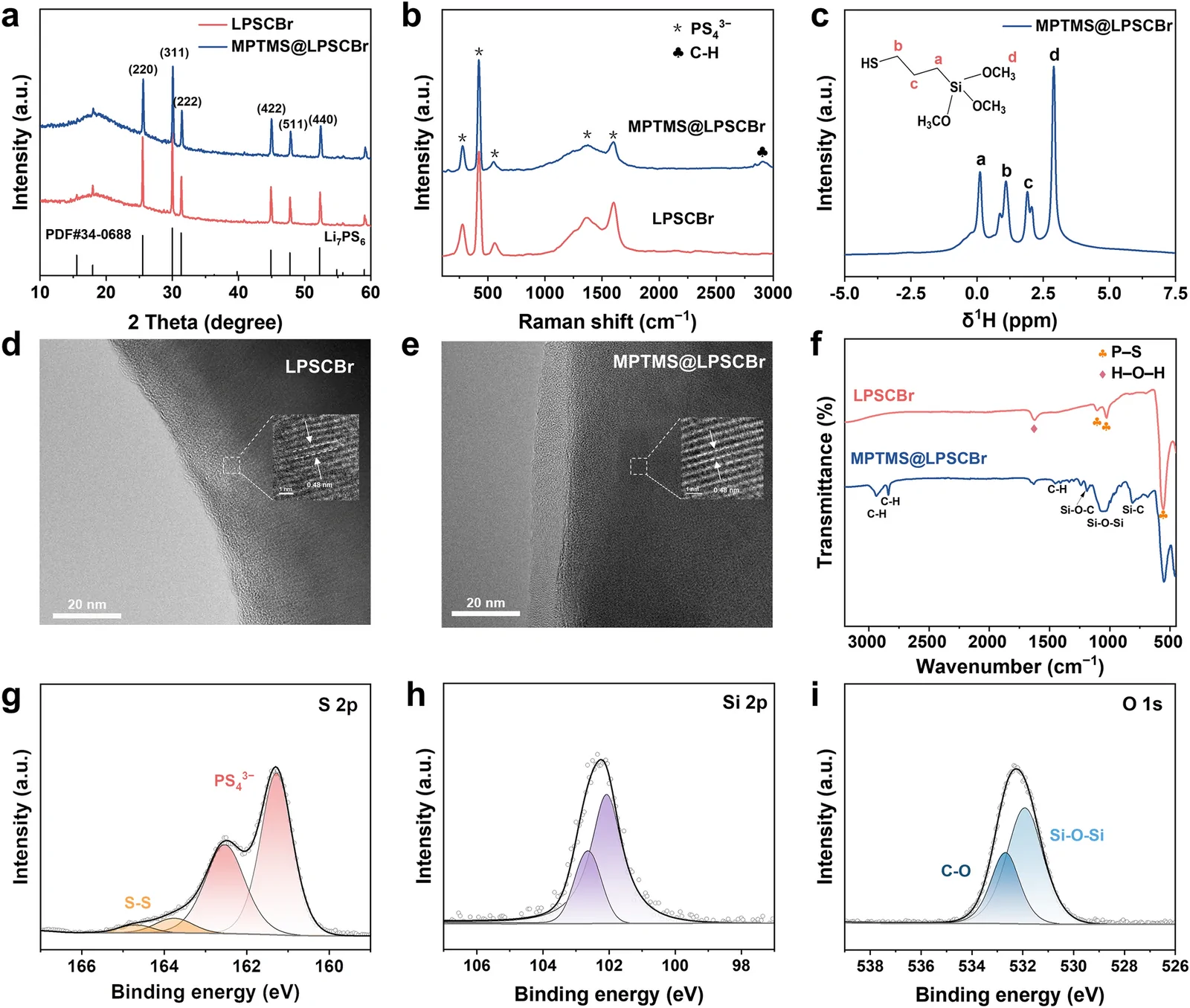
Structural and chemical characterization of LPSCBr and MPTMS@LPSCBr electrolytes. **a** XRD patterns of LPSCBr and MPTMS@LPSCBr. **b** Raman spectra of LPSCBr and MPTMS@LPSCBr. **c** Solid-state ^1^H NMR spectrum of MPTMS@LPSCBr. **d** HRTEM images of bare LPSCBr.** e** HRTEM images of MPTMS@LPSCBr. **f** FTIR spectra of LPSCBr and MPTMS@LPSCBr. XPS spectra of MPTMS@LPSCBr: **g** S 2*p*, **h** Si 2*p*, and **i** O 1*s* spectra

The representative LPSCBr was prepared via solid-state reaction method [27, 28]. As shown in Fig. 1a, the XRD pattern of LPSCBr matches well with the cubic argyrodite structure. Rietveld refinement was performed to verify its phase purity and structural integrity (Fig. S1), and the refined results confirm a pure cubic argyrodite phase. XRD analysis demonstrates that the bulk crystal structure of LPSCBr is well preserved after MPTMS coating, with no observable phase transitions or peak broadening. As presented in Fig. 1b, the Raman spectrum of MPTMS@LPSCBr shows a characteristic C–H vibrational mode at 2906 cm^−1^, verifying the successful anchoring of MPTMS.

Solid-state ^1^H NMR spectroscopy was used to identify hydrogen-containing functional groups on MPTMS@LPSCBr (Fig. 1c). In the ^1^H NMR spectrum of MPTMS@LPSCBr, multiple peaks are assigned to characteristic protons of MPTMS: The peak at δ≈0.1 ppm (peak a) is attributed to methylene protons adjacent to silicon (Si–CH_2_^−^); δ≈1.0 ppm (peak b) corresponds to methylene protons near the mercapto group (HS–CH_2_^−^); δ≈1.5 ppm (peak c) is assigned to the middle methylene group (–CH_2_–); δ≈3.5 ppm (peak d) is identified as methoxy protons (–OCH_3_) attached to silicon. The clear identification of these MPTMS-derived peaks confirms the successful and stable coating of MPTMS on LPSCBr, as well as the effectiveness of the interface modification. The ^31^P solid-state NMR spectrum (Fig. S2) shows that the PS_4_^3−^ tetrahedral coordination (at 85.3 ppm) is well retained in MPTMS@LPSCBr, with no detectable phosphorus oxide species, indicating negligible structural degradation of the electrolyte during mechanochemical processing.

The microstructure and elemental composition of MPTMS@LPSCBr samples were characterized by SEM images and elemental mapping in Fig. S3. Uniform Si distribution confirms successful MPTMS grafting. Low-magnification SEM of cold-pressed pellets (Fig. S4) further verifies that MPTMS modification barely changes the compacted surface morphology and powder processability compared with pristine LPSCBr. High-magnification SEM coupled with EDS mapping of the pellet cross section (Fig. S5) demonstrates evenly dispersed Si species within the solid electrolyte bulk. In Fig. 1d–e, HRTEM imaging reveals a continuous amorphous coating layer (≈10 nm thick) conformally covering LPSCBr particles, distinct from the crystalline lattice fringes of bare LPSCBr (lattice spacing of 0.48 nm, corresponding to the (200) plane). Thermogravimetric analysis (TGA) was conducted to quantify the coating content. This weight loss feature corresponds to the thermal decomposition of organic segments in MPTMS. Bare LPSCBr retains 80.8% of its initial mass at 800 °C, whereas MPTMS@LPSCBr shows a lower mass retention of 70.9% (Fig. S6). Further TGA profiles of 20:1 and 5:1 formulations (Fig. S7) display gradually decreasing residual weights as MPTMS dosage increases. The difference in thermal stability arises from the degradation and volatilization of MPTMS’s organic segments during heating, introducing extra weight loss compared with the thermally stable inorganic LPSCBr matrix.

The chemical bonding features of bare LPSCBr and MPTMS@LPSCBr are characterized by FTIR spectra in Fig. 1f. Inherent peaks related to S-containing groups in LPSCBr are observed, such as those at ~555.8, 1027.4, and 1107.4 cm^−1^ (attributed to P–S bond vibrations of PS_4_^3−^). MPTMS@LPSCBr exhibits distinct absorption bands: Peaks at ~2934.5 and ~2838.2 cm^−1^ are assigned to C–H stretching of the propyl chain, the band at ~1047.2 cm^−1^ corresponds to asymmetric stretching of Si–O–Si linkages, and the band at ~1191.3 cm^−1^ is assigned to Si–O–C stretching, providing further evidence for a chemical interaction between MPTMS and the LPSCBr surface. A broad absorption at ~1623 cm^−1^ is assigned to H–O–H bending from adsorbed water.

To clarify the chemical bonding states of MPTMS@LPSCBr, XPS was conducted. In the S 2*p* spectrum (Fig. 1g), besides the dominant peak from the PS_4_^3−^ framework, distinct peaks at 163.8 and 164.7 eV corresponding to S–S bonds are observed, verifying covalent interaction between thiol (–SH) groups of MPTMS and surface sulfur sites of LPSCBr. The Si 2*p* spectrum (Fig. 1h) shows characteristic Si peaks that can be deconvoluted into several components, indicating successful MPTMS grafting and formation of Si-containing species such as siloxane. For the O 1*s* spectrum (Fig. 1i), deconvoluted peaks correspond to C–O and Si–O–Si species. The presence of Si–O–Si bonds further confirms a cross-linked siloxane network from hydrolysis and condensation of methoxysilane groups.

### Effectiveness of MPTMS Buffering Layer

The Nyquist plots of bare LPSCBr and MPTMS-coated LPSCBr are shown in Figs. S8 and S9. For bare LPSCBr, a nearly straight line is observed, indicating minimal grain boundary resistance, with a high Li⁺ conductivity of 13 mS cm^−1^ at 25 °C. The Nyquist plot of MPTMS@LPSCBr suggests a slight increase in grain boundary resistance due to the MPTMS coating layer. Remarkably, the Li⁺ conductivity only decreases by 16.9%, from 13 to 10.8 mS cm^−1^, which corresponds to 83.1% retention of the original value. To gain further insight into the origin of this preserved conductivity, the ^7^Li spin echo solid-state NMR spectra of LPSCBr and MPTMS@LPSCBr were collected (Fig. S10). The nearly identical chemical shifts imply that the Li^+^ chemical environment remains unaltered upon MPTMS coating. Meanwhile, the similar peak widths indicate negligible interference of the ultrathin MPTMS layer on bulk Li^+^ transport, which is consistent with the minimal ionic conductivity reduction, verifying that the coating maintains Li^+^ mobility while achieving surface modification. However, MPTMS-coated LPSCBr with a 5:1 (LPSCBr/MPTMS) mass ratio shows the highest impedance, indicating a trade-off between MPTMS content and ionic transport (excessive MPTMS hinders Li⁺ conduction). The MPTMS-coated LPSCBr with a 5:1 mass ratio delivers an ionic conductivity of 7.24 mS cm^−1^ at 25 °C. By contrast, the 20:1 counterpart achieves a higher ionic conductivity of 11.8 mS cm^−1^ at 25 °C owing to insufficient silane loading and thinner discontinuous coating.

**Fig. 2.**
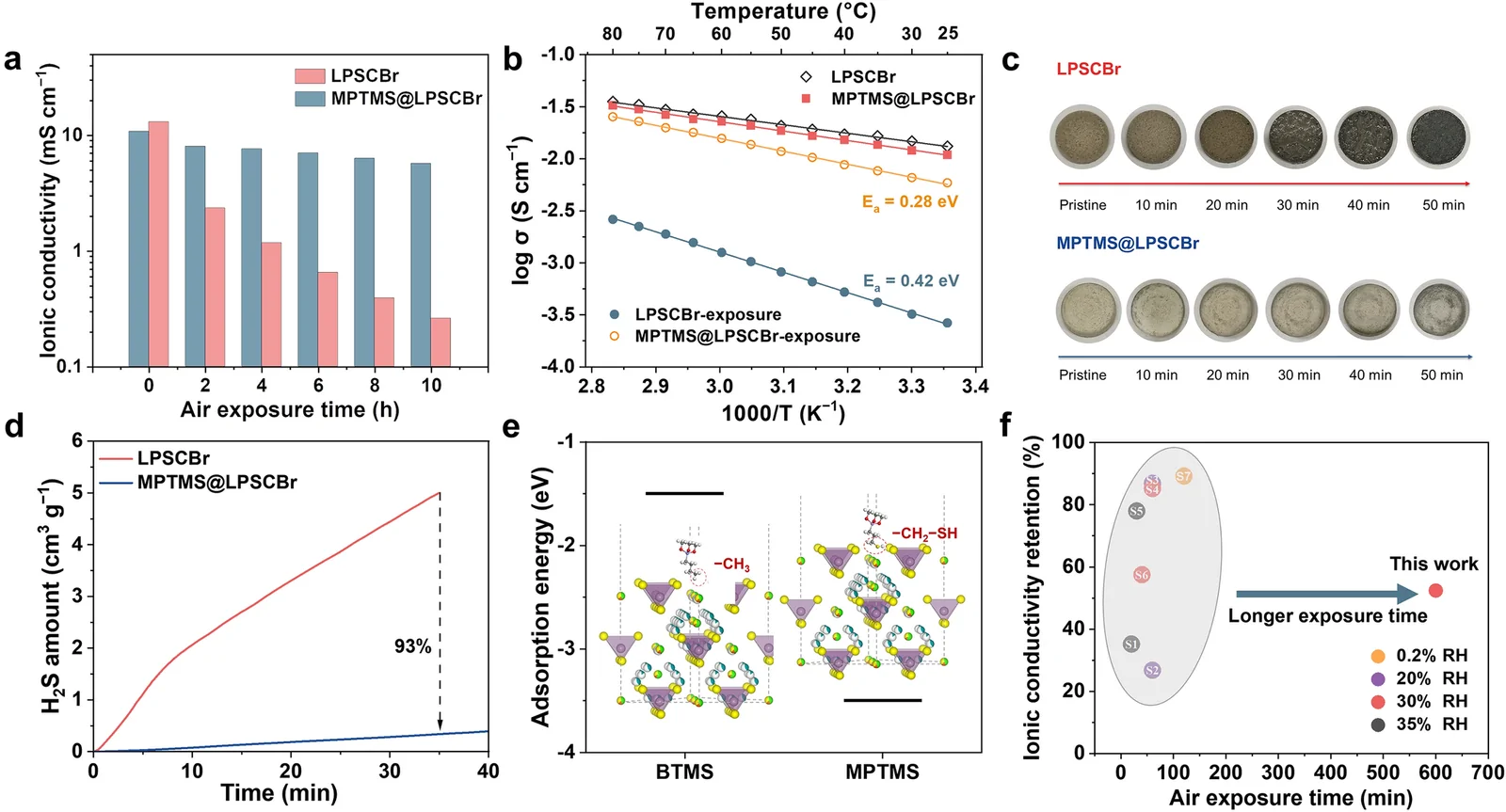
Characterization of LPSCBr and MPTMS@LPSCBr after exposure to humid ambient environments.** a** Ionic conductivity of LPSCBr and MPTMS@LPSCBr as a function of air exposure time at 30% RH. **b** Arrhenius plots of ionic conductivity for bare LPSCBr, MPTMS@LPSCBr, and their air-exposed counterparts. **c** Optical photographs of LPSCBr and MPTMS@LPSCBr samples at various air exposure times (pristine, 10 min, 20 min, 30 min, 40 min, and 50 min). **d** Time-dependent H_2_S evolution amount from LPSCBr and MPTMS@LPSCBr upon air exposure (inset: H_2_S suppression ratio at 35 min). **e** Calculated adsorption energy profiles and schematic illustrations for BTMS and MPTMS on the LPSCBr surface. **f** Normalized ionic conductivity retention of samples versus air exposure time under different relative humidity conditions, with the result of this work highlighted

The ionic conductivity of bare and MPTMS@LPSCBr is plotted against exposure time to 30% RH (25 °C) in Fig. 2a. The ionic conductivity of bare LPSCBr decreases rapidly from 13 to 2.34 mS cm^−1^ after 2 h and 0.26 mS cm^−1^ after 10 h, due to severe PS_4_^3−^ hydrolysis. In contrast, MPTMS@LPSCBr maintains 8.0 mS cm^−1^ after 2 h and 5.7 mS cm^−1^ after 10 h. Arrhenius fitting (25–80 °C, Fig. 2b) shows that as-prepared MPTMS@LPSCBr has an activation energy (E_a_) of 0.28 eV, nearly identical to bare LPSCBr (0.27 eV). After 10-h humid air exposure, the E_a_ of MPTMS@LPSCBr only rises to 0.30 eV, while bare LPSCBr E_a_ jumps to 0.42 eV, confirming the MPTMS coating shields Li^+^ transport pathways from moisture degradation.

Figure 2c displays the optical photographs of bare LPSCBr and MPTMS@LPSCBr samples at various air exposure times (bare, 10, 20, 30, 40, and 50 min). Bare LPSCBr undergoes a distinct color change from its initial pale appearance to dark gray/black within 50 min, visually reflecting severe structural degradation induced by moisture absorption (e.g., hydrolysis and formation of secondary phases). In contrast, MPTMS@LPSCBr retains its original light color even after 50 min of air exposure, directly demonstrating the effective moisture shielding by the MPTMS coating. This macroscopic observation corroborates the aforementioned electrochemical results, providing direct evidence for the enhanced air stability of MPTMS-modified LPSCBr. The chemical stability of as-prepared sulfide electrolytes against moisture-induced degradation was quantitatively evaluated by monitoring H_2_S generation during controlled air exposure. Bare LPSCBr and MPTMS-coated LPSCBr powders were exposed to controlled ambient air (25 °C, 30% RH). As shown in Fig. 2d, the H_2_S release of bare LPSCBr reaches 5 cm^3^ g^−1^ after 35 min of exposure, indicative of severe hydrolysis-driven structural decomposition. In stark contrast, the MPTMS-coated LPSCBr with a 10:1 mass ratio (LPSCBr/MPTMS) exhibits a drastically reduced H_2_S emission of 0.33 cm^3^ g^−1^, corresponding to 93.0% suppression efficiency. The H_2_S evolution for MPTMS@LPSCBr variants with 5:1 and 20:1 mass ratios under the same conditions was quantified in Fig. S11: The H_2_S emission decreases to 0.31 cm^3^ g^−1^ for the 5:1 ratio and to 0.55 cm^3^ g^−1^ for the 20:1 ratio. Taken together, these findings indicate that MPTMS@LPSCBr with a 10:1 mass ratio represents the optimal formulation, as it strikes an ideal balance between enhanced air stability (via effective moisture resistance and suppressed H_2_S evolution) and uncompromised ionic transport properties (with minimal conductivity loss and preserved Li^+^ diffusion kinetics).

To comprehensively elucidate the interfacial bonding superiority of MPTMS, we first turn to density functional theory (DFT) calculations and structural characterizations. As illustrated in Fig. S12, the optimized atomic structures reveal distinct molecular architectures: Trimethoxymethylsilane (MTMS) features a short methyl chain, butyltrimethoxysilane (BTMS) incorporates a longer butyl chain, while MPTMS introduces a thiol (–SH) group. Calculations demonstrate a pronounced adsorption energy enhancement for MPTMS (−3.52 eV) relative to the thiol-free reference system (−1.48 eV), quantitatively confirming the essential contribution of terminal –SH groups. The –SH group forms strong covalent S–S bonds with sulfur sites on the LPSCBr surface, stabilizing the PS_4_^3−^ tetrahedral framework and inhibiting its hydrolysis.

To validate these theoretical insights experimentally, we systematically monitored H_2_S evolution for MPTMS-, BTMS-, and MTMS-coated LPSCBr samples (LPSCBr/hydrophobic molecules = 10:1 mass ratio). As shown in Fig. S13, MPTMS@LPSCBr exhibits a drastically suppressed H_2_S release of only 0.39 cm^3^ g^−1^ at 40 min, while BTMS-coated counterparts (with a comparable long alkyl chain but lacking the thiol group) show a higher emission of 1.27 cm^3^ g^−1^ under the same conditions, and this stark contrast directly proves the pivotal role of the –SH group in blocking hydrolytic degradation. Furthermore, for MTMS-coated LPSCBr (which features a short methyl chain and no –SH group), the H_2_S release rises to 3.288 cm^3^ g^−1^ at 40 min. This comparison not only emphasizes the irreplaceable contribution of the –SH group but also reveals that longer alkyl chains (as in BTMS) can moderately enhance moisture resistance.

The bifunctional MPTMS coating strategy possesses broad universality for thiophosphate electrolytes, since the covalent S–S bonding and silanol condensation reactions merely occur on particle surfaces without damaging bulk P–S or Ge–S lattices. Taking typical Li_10_GeP_2_S_12_ (LGPS) as a representative verification system, we carried out identical ball-milling grafting treatment under the same parameters as LPSCBr. After modification, the H_2_S release of LGPS is sharply suppressed by 91% within 35 min humid exposure (Fig. S14), which directly proves that this mild mechanochemical route does not destroy the crystal skeleton of sulfide electrolytes. This confirms that our coating method can be extended to various mainstream superionic sulfide solid electrolytes.

To further benchmark the air stability of our MPTMS@LPSCBr electrolyte against state-of-the-art sulfide solid electrolytes, we compared the normalized ionic conductivity retention under different relative humidity conditions with previously reported studies, as presented in Fig. 2f and Table S1. The comparison reveals that most reported works only achieve short-term air stability under low-humidity conditions, while our MPTMS@LPSCBr electrolyte maintains favorable ionic conductivity retention even after 600 min of exposure at 30% RH, representing one of the best comprehensive air-stability performances among reported sulfide electrolyte systems.

### Interaction Characterization and Mechanical Properties

Furthermore, we systematically characterized the moisture stability, structural integrity, and surface chemical state of MPTMS@LPSCBr, with bare LPSCBr as a control, using dynamic humidity exposure tests, XRD, Raman, and XPS.

**Fig. 3:**
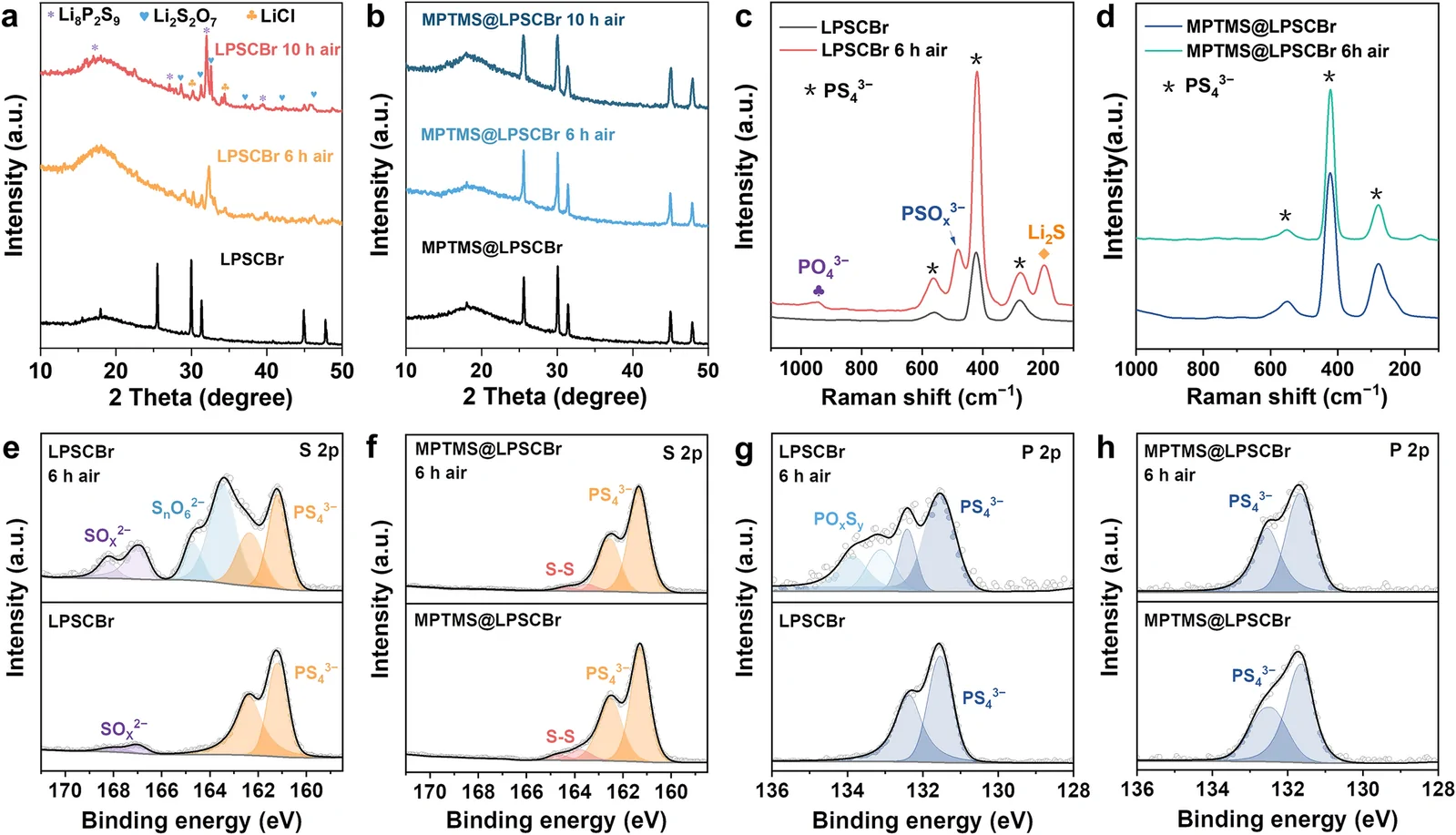
Characterization of LPSCBr and MPTMS@LPSCBr samples with different humid air exposure times. **a, b** XRD patterns obtained for LPSCBr and MPTMS@LPSCBr samples exposed to humid air for up to 10 h. **c, d** Raman spectra collected on LPSCBr and MPTMS@LPSCBr samples exposed to humid air for up to 6 h. **e, f** High-resolution S 2*p* XPS spectra, and **g, h** P 2*p* XPS spectra obtained for LPSCBr and MPTMS@LPSCBr samples exposed to humid air for up to 6 h

To assess the impact of MPTMS modification on crystalline integrity, XRD patterns of bare LPSCBr and MPTMS@LPSCBr were collected after 6 and 10 h of ambient air exposure (Fig. 3a, b). Pristine bare LPSCBr shows well-resolved diffraction peaks assigned to cubic argyrodite, which features ordered PS_4_^3−^ tetrahedra responsible for fast Li^+^ conduction. After 6 h of air aging, the characteristic argyrodite diffraction signals almost vanish, accompanied by strong impurity peaks at 2θ = 29.1°, 31.4°, and 32.3°, corresponding to poorly conductive Li_8_P_2_S_9_ and Li_2_S_2_O_7_. Broad peaks originating from LiCl degradation emerge at 30.2° and 34.5°. Upon extending exposure to 10 h, the intensities of all impurity peaks rise dramatically, revealing severe structural collapse of the argyrodite framework. In stark contrast, MPTMS@LPSCBr exhibits superior structural stability: its argyrodite diffraction peaks remain sharp after 6 h of aging without detectable impurity signals (Fig. 3b), and the key characteristic peaks are still well maintained even after 10 h of air exposure.

Raman spectroscopy was employed to investigate the structural stability of both samples under controlled ambient air exposure. The characteristic band at ~424 cm^−1^ originates from PS_4_^3−^ units, which dominate Li^+^ conduction in sulfide electrolytes (Fig. 3c). After 6 h of air aging, this peak of bare LPSCBr shifts distinctly toward lower wavenumbers, demonstrating partial cleavage of P–S bonds and the generation of oxy-thiophosphate PSO_x_^3−^. Extra vibration bands at 197.0 cm^−1^ (Li_2_S) and 944.1 cm^−1^ (PSO_x_^3−^) are clearly observed for degraded bare LPSCBr. By comparison, MPTMS@LPSCBr displays negligible shift of the PS_4_^3−^ peak with no detectable impurity bands, verifying that the MPTMS coating effectively suppresses moisture-triggered structural degradation (Fig. 3d).

To unravel the chemical mechanism of moisture resistance, high‑resolution XPS spectra of S 2*p* and P 2*p* were collected for bare LPSCBr and MPTMS@LPSCBr after 6h air exposure (Fig. 3e–h). The S 2*p* spectrum of bare LPSCBr shows three main components: the dominant conductive PS_4_^3−^ phase, S_n_O_6_^2−^ from partial decomposition, and strong SO_x_^2−^ signals from hydrolytic oxidation. The drastically reduced PS_4_^3−^ intensity and intense S_n_O_6_^2−^ and SO_x_^2−^ peaks confirm severe surface degradation. For MPTMS@LPSCBr, the SO_x_^2−^ peak at 167.0 eV is almost absent, demonstrating that MPTMS effectively inhibits P–S bond cleavage and sulfur oxidation. The hydrophobic MPTMS layer lowers moisture adsorption, and covalent bonding stabilizes surface PS_4_^3−^ against hydrolysis. The P 2*p* spectra further support this protection. Bare LPSCBr displays strong PO_x_S_y_ peaks at ~133.1 and 133.8 eV, arising from P–S bond hydrolysis and oxygen substitution. In contrast, MPTMS@LPSCBr retains the dominant PS_4_^3−^ peaks at 131.7 and 132.5 eV with no observable PO_x_S_y_ signals, confirming that MPTMS prevents P–O formation and maintains the conductive P environment.

To further distinguish the independent degradation induced by pure moisture hydrolysis from the synergistic oxidation of H_2_O and O_2_, supplementary XRD characterizations under oxygen-free humid N_2_ atmosphere (30% RH, 25 °C) were performed, and the corresponding patterns are displayed in Figs. S15 and S16. For bare LPSCBr aged under O_2_-free humid N_2_ for 2 h (Fig. S15), only hydrolysis-derived impurities including Li_3_PO_4_ and LiOH are detected, while characteristic diffraction peaks of crystalline Li_2_S_2_O_7_ are completely absent. When the aging time is prolonged to 10 h (Fig. S16), the intensities of Li_3_PO_4_ and LiOH increase significantly, and hydrated LiCl·H_2_O emerges as an extra hydrolysis product. Even after long-term oxygen-free humid exposure, no Li_2_S_2_O_7_ oxidation by-product can be identified across the full 2θ range.

The significant difference in degradation products between oxygen-free humid conditions and ambient air can be rationalized based on established single-component atmospheric reaction mechanisms for sulfide electrolytes [29–31]. As a chemically inert gas, N_2_ does not participate in any lattice decomposition or redox reaction with LPSCBr. Dry CO_2_ exhibits only negligible surface reactivity, forming merely amorphous Li_2_CO_3_ thin layers without crystalline sulfate by-products. Under anhydrous pure oxygen atmosphere, the intact PS_4_^3−^ tetrahedral framework possesses excellent oxidation resistance; only minor amorphous PO_x_S_y_ surface species are generated, while crystalline high-valent Li_2_S_2_O_7_ cannot form.

In oxygen-free humid environments, moisture exclusively induces hydrolysis of LPSCBr by breaking P–S covalent bonds. The fractured phosphorus and sulfur fragments recombine with lithium ions and water molecules to form hydrolysis products, including LiOH, Li_3_PO_4_, and hydrated LiCl·H_2_O. Since no external oxidizing agent is available, the undercoordinated sulfur defects produced during hydrolysis remain chemically stable and cannot be further oxidized into high-valent sulfate species. Consequently, pure humid hydrolysis only triggers structural corrosion without generating crystalline Li_2_S_2_O_7_.

In sharp contrast, ambient air contains both moisture and abundant oxygen, enabling a synergistic hydrolysis–oxidation degradation pathway. Water molecules first initiate lattice hydrolysis and generate numerous reactive sulfur defect sites. These defect sites are thermodynamically unstable under aerobic conditions and are readily oxidized by oxygen, finally producing large amounts of crystalline Li_2_S_2_O_7_ alongside conventional hydrolysis by-products.

Collectively, by combining literature single-gas conclusions and our controlled XRD experiments, we successfully differentiated two independent degradation modes of LPSCBr. Pure water vapor leads to simple hydrolysis and mixed inorganic impurities, whereas the coexistence of H_2_O and O_2_ introduces an additional oxidative pathway responsible for high-valent Li_2_S_2_O_7_ formation. Therefore, the severe structural degradation and crystalline sulfate generation during air exposure originate from the coupling effect of hydrolysis and secondary oxidation, rather than water hydrolysis alone.

### Electrochemical Performance of Air-Exposed MPTMS@LPSCBr in All-Solid-State Cells

To verify the suitability of the MPTMS@LPSCBr electrolyte, two types of ASSLBs were assembled using NCM83 as the composite cathode and lithium–indium alloy as the anode. The LPSCBr and MPTMS@LPSCBr powders were stored in sealed containers at 30% RH for 2 h before being used as the electrolyte layer. The assembled cells were tested at 25 °C within a voltage range of 2.7–4.3 V (vs. Li^+^/Li).

**Fig. 4:**
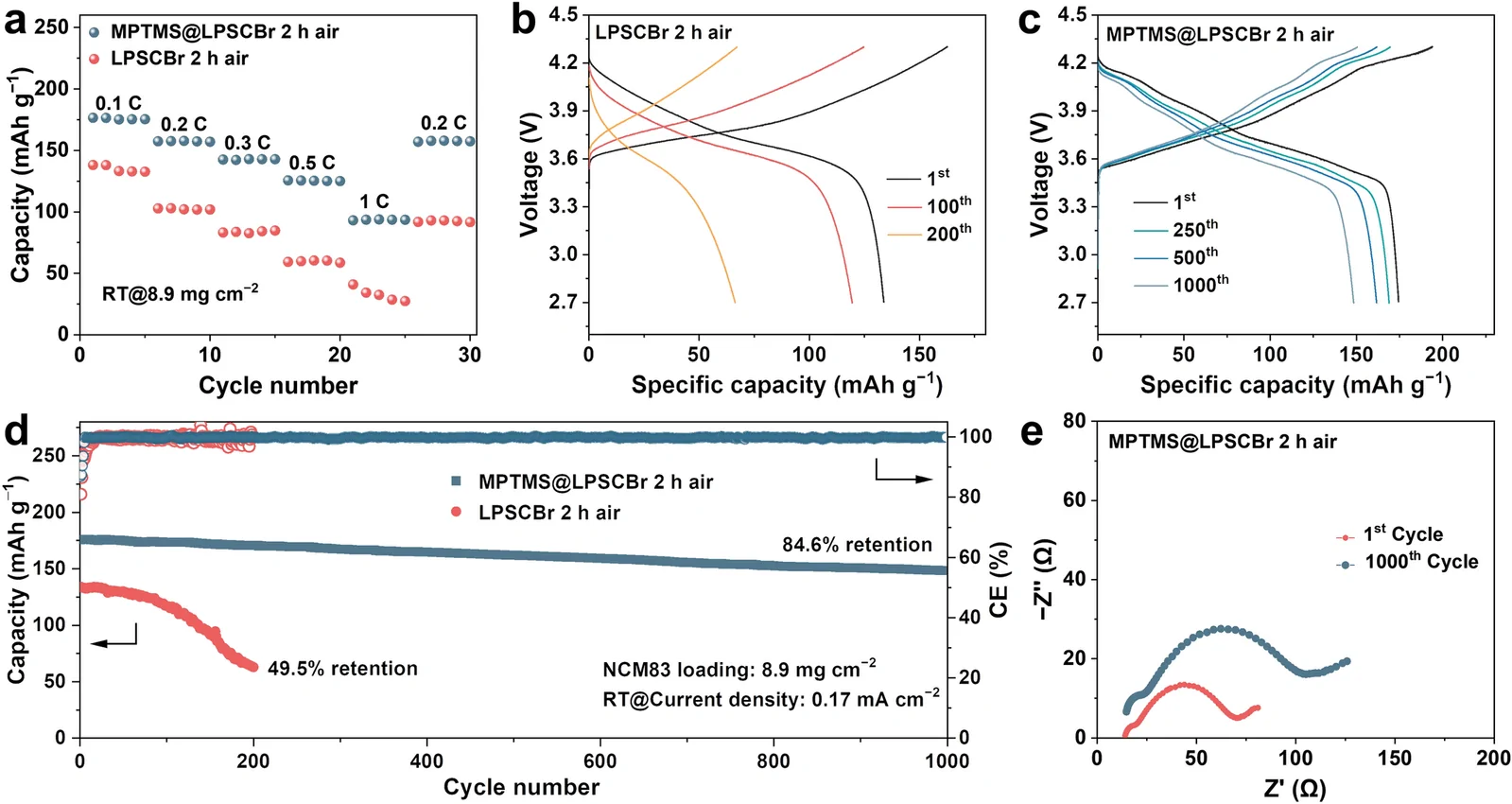
Electrochemical performance of LPSCBr and MPTMS@LPSCBr after 2 h of 30% RH air exposure. **a** Rate capabilities of ASSLBs using MPTMS@LPSCBr and LPSCBr electrolytes, tested at 25 °C with current densities ranging from 0.1 C to 1 C. **b** Galvanostatic charge/discharge curves of LPSCBr ASSLBs at 0.1 C with different cycles. **c** Galvanostatic charge/discharge curves of MPTMS@LPSCBr-based ASSLBs at 0.1 C with different cycles. **d** Long-term cycling stability of the ASSLBs at 25 °C, with a cathode areal mass loading of 8.9 mg cm^−2^ and a current density of 0.17 mA cm^−2^. **e** Evolution of interfacial resistance for MPTMS@LPSCBr-based cells over 1000 cycles

The charge/discharge curves at different current densities are shown in Figs. S17 and S18. As shown in Fig. 4a, the MPTMS@LPSCBr-based cell outperforms the bare LPSCBr-based cell across all tested current densities (0.1 to 1 C). At current rates of 0.1, 0.2, 0.3, 0.5, and 1 C, the MPTMS@LPSCBr cell delivers reversible capacities of 176.5, 157.4, 142.5, 125.6, and 93.1 mAh g^−1^, respectively. By contrast, the bare LPSCBr cell exhibits much lower reversible capacities of 138.1, 102.7, 83.2, 59.3, and 40.9 mAh g^−1^ at the corresponding current densities. This enhanced rate capability is attributed to the effective moisture-blocking MPTMS coating, which preserves the structural and chemical integrity of the LPSCBr electrolyte and thus maintains high Li^+^ conductivity.

Long-term cycling tests (Fig. 4d) further confirm the superior air and electrochemical stability of the MPTMS-modified electrolyte. The Coulombic efficiency (CE) evolution during cycling directly reflects the reversibility of interfacial electrochemical reactions. After 2 h of air exposure, the bare LPSCBr cell delivers an initial Coulombic efficiency (ICE) of only 81.0%, and its CE rises slowly with cycling, remaining below 99% even after dozens of cycles. This low and gradually climbing CE is associated with continuous irreversible side reactions at the interface, driven by hydrolytic products of the sulfide electrolyte. In sharp contrast, the MPTMS@LPSCBr cell exhibits a much higher ICE of 86.8% under identical air exposure conditions, and its CE rapidly increases to above 99% within the initial 5 cycles and then stabilizes at approximately 99.6% throughout the subsequent 1000 cycles. This highly reversible and stable CE behavior verifies that the MPTMS coating effectively suppresses electrolyte hydrolysis and inhibits persistent interfacial parasitic reactions.

In terms of capacity retention, the MPTMS@LPSCBr cell maintains 84.6% of its initial capacity after 1000 cycles at 0.1 C, while the exposed bare LPSCBr cell retains only 49.5% of its capacity after 200 cycles. The galvanostatic charge/discharge profiles are shown in Fig. 4b, c. Notably, the NCM83 cell using the MPTMS@LPSCBr electrolyte shows lower overpotentials than the LPSCBr cell, which is attributed to the well-preserved Li^+^ conductivity. The retained structural integrity of MPTMS@LPSCBr minimizes ionic transport resistance, whereas bare LPSCBr suffers from partial hydrolysis that disrupts its argyrodite framework and reduces conductivity.

The impedance data in Fig. 4e provide direct evidence of improved interfacial stability with MPTMS modification. Over 1000 cycles, MPTMS@LPSCBr-based cells show a modest increase in interfacial resistance from ~40 to ~101 Ω. However, bare LPSCBr-based cells exhibit a dramatic rise from 442 to ~1279 Ω after 50 cycles (Fig. S19). These results unequivocally demonstrate that the MPTMS modification strategy successfully confers exceptional air stability to the sulfide electrolyte, which directly translates into superior electrochemical stability in practical all-solid-state batteries.

### Enhancing Air Stability and Electrochemical Performance of Sulfide-Based Solid Electrolytes via MPTMS Modification

Beyond the air stability of MPTMS@LPSCBr verified in the last section, the interfacial instability between sulfide-based solid electrolytes and high-voltage cathode materials remains a critical challenge [32, 33]. Inspired by the wide utility of trimethylsilyl functional materials for interface regulation in liquid and solid batteries [34], we developed an MPTMS surface-coating strategy to construct a protective siloxane layer on LPSCBr solid electrolyte particles. Pre-synthesized MPTMS@LPSCBr powder was fabricated at a fixed LPSCBr/MPTMS mass ratio of 10:1. All modified cells adopt MPTMS@LPSCBr electrolyte, and the composite cathode was composed of NCM83, MPTMS@LPSCBr, and VGCF (70:30:1 by mass), with no free MPTMS powder blended into electrodes.

**Fig. 5:**
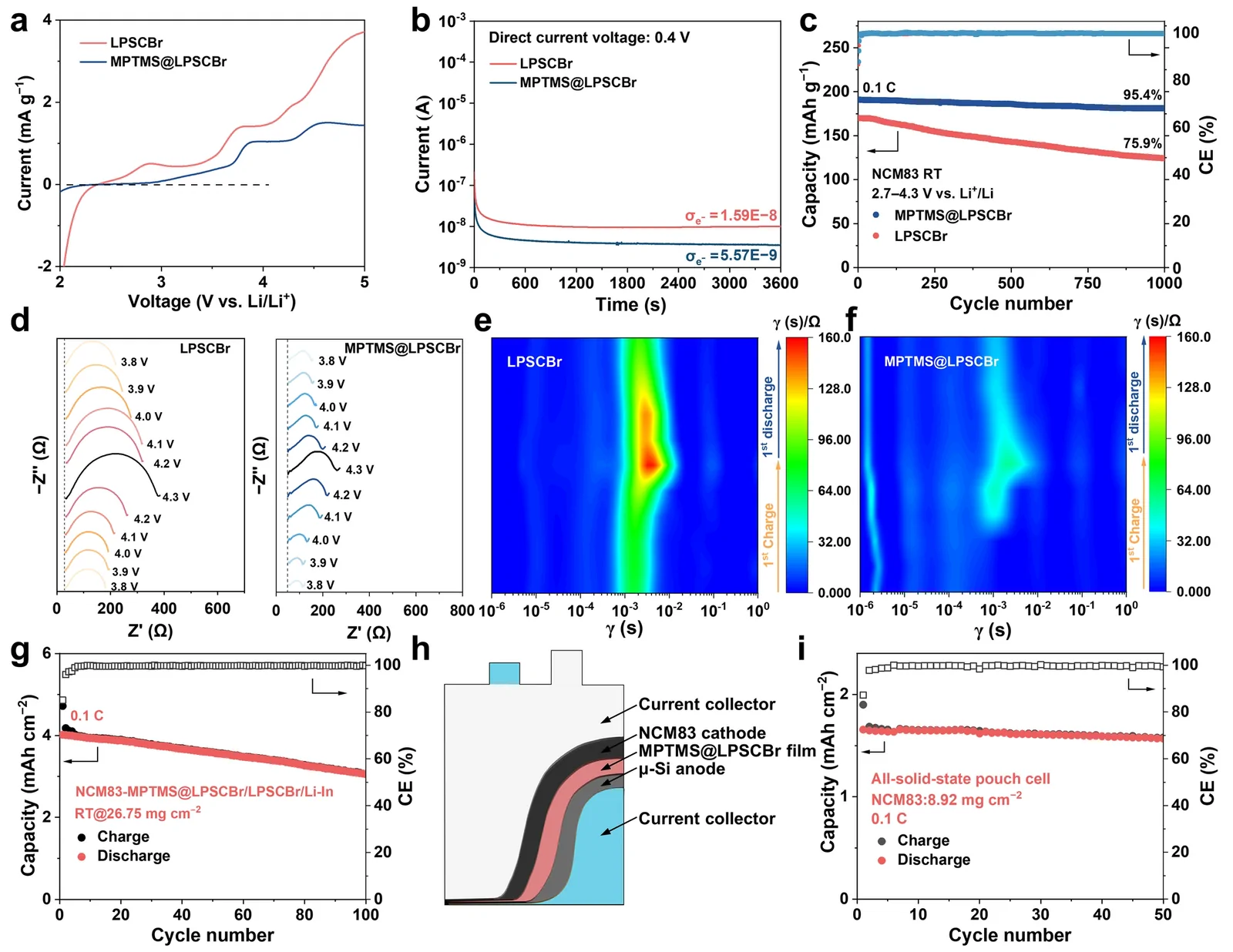
**a** Cyclic voltammetry curves of pristine LPSCBr and MPTMS@LPSCBr. **b** Current–time curves of bare LPSCBr and MPTMS@LPSCBr powders measured under a constant direct current voltage of 0.4 V. **c** Cycling performance and coulombic efficiency of all-solid-state NCM83/LPSCBr/Li-In cells, where the composite cathodes were blended with either bare LPSCBr or MPTMS@LPSCBr powder, tested at 0.1 C and 25 °C within 2.7–4.3 V (vs. Li/Li⁺). **d** In situ EIS profiles recorded over the first charge–discharge cycle for cells whose composite cathodes contain bare LPSCBr or MPTMS@LPSCBr powder, along with the corresponding 3D DRT plots (**e, f**). **g** Long-term cycling performance and Coulombic efficiency of high-areal-loading all-solid-state cells with the configuration NCM83-MPTMS@LPSCBr/LPSCBr/Li-In at 0.1 C and 25 °C. **h** Schematic diagram of the NCM83-MPTMS@LPSCBr/LPSCBr film/μ-Si full electrode structure. **i** Cycling performance and Coulombic efficiency of the all-solid-state pouch cell assembled with NCM83 cathode (areal loading: 8.92 mg cm^−2^) at 0.1 C

To evaluate the effect of MPTMS surface coating on the electrochemical oxidation stability of LPSCBr, cyclic voltammetry (CV) measurements were first performed. As shown in Figs. 5a and S20, the bare LPSCBr electrode exhibits a continuously increasing oxidation current above approximately 2.58 V (vs. Li⁺/Li), which is associated with the gradual decomposition of the electrolyte. In contrast, the MPTMS@LPSCBr electrode demonstrates a significantly suppressed current density throughout the scanned voltage window (Fig. S21). The MPTMS@LPSCBr sample exhibits drastically suppressed parasitic Faradaic currents across the full voltage window, and its oxidation onset potential shifts positively to ~2.95 V. This positive shift of oxidative decomposition potential directly confirms the improved anodic stability of LPSCBr after surface coating.

Beyond improved electrochemical oxidation stability, the conformal MPTMS coating effectively reduces the electronic conductivity of LPSCBr. A clear quantitative correlation between coating thickness/loading and insulating performance is established across LPSCBr/MPTMS ratios of 20:1, 10:1, and 5:1 (Figs. 5b and S22). Electronic conductivity decreases monotonically with rising MPTMS loading: Bare LPSCBr reaches 1.59 × 10^−8^ S cm^−1^, while the balanced 10:1 MPTMS@LPSCBr drops to 5.57 × 10^−9^ S cm^−1^. The cross-linked Si–O–Si siloxane network is electronically insulating and forms a continuous barrier on electrolyte grains, blocking leakage electrons and inhibiting high-voltage parasitic redox reactions between sulfide electrolyte and high-NCM cathode. The 10:1 coating density realizes the best balance between electron insulation and unimpeded Li^+^ transport, effectively alleviating sulfide electrolyte decomposition during cycling.

Furthermore, galvanostatic cycling tests reveal that the MPTMS@LPSCBr electrolyte delivers an initial discharge specific capacity as high as 190.8 mAh g^−1^, with its capacity retention reaching an impressive 95.4% after 1000 cycles, manifesting superior cycling stability and capacity retention in comparison with the cathode matched with bare LPSCBr electrolyte (Fig. 5c). The voltage profiles during cycling show reduced polarization and more stable operation, indicating improved interfacial compatibility with the NCM83 cathode (Fig. S23). In situ EIS analysis further corroborates these findings: MPTMS-modified samples exhibit lower interfacial resistance under high voltage and more stable impedance evolution characteristics during cycling (Fig. 5d, e). The relationship between impedance changes in electrochemical processes and battery degradation was analyzed using the distributed relaxation time (DRT) technique [35, 36]. Six peaks (D1 to D6) were identified in the DRT spectrum (Figs. S24 and S25), each corresponding to distinct electrochemical processes. D1–D3 in the high-frequency range reflect Li^+^ transport across the current collector/Li-In interface, Li-In bulk phase, and sulfide solid electrolyte interlayer, respectively. D4–D5 correspond to charge transfer at the NCM83 interface, while D6 reflects Li-In anode lithiation/delithiation [37]. The DRT profile of the MPTMS-modified cell demonstrates a remarkably higher degree of consistency and reversibility throughout the charge–discharge cycle (Fig. 5f). The minimal shift and highly reversible nature of the D4 and D5 peaks, which are associated with charge transfer at the NCM83 interface, directly verify the enhanced interfacial compatibility and electrochemical reversibility afforded by the MPTMS modification. Complementary postmortem XPS results (Figs. S26 and S27) further validate this protection effect. Bare LPSCBr generates abundant decomposition by-products after short cycling, while MPTMS@LPSCBr retains intact Si–O–Si and S–S bonding without obvious degradation over long cycles, confirming the siloxane barrier suppresses cross-interfacial redox reactions. Under high loading conditions of 26.75 mg cm^−2^, the NCM83-MPTMS@LPSCBr/LPSCBr/Li-In battery still exhibits outstanding cycling stability (Figs. 5g and S28). After 100 cycles at a 0.1 C rate, the battery maintains a capacity retention rate of 76.1%.

Furthermore, we prepared the MPTMS@LPSCBr electrolyte membrane via a wet-coating method. Pre-synthesized MPTMS@LPSCBr powder and polyisobutylene binder solution were co-ball-milled to form homogeneous slurry during film fabrication. The slurry was cast onto a release polyethylene terephthalate film using a doctor blade and calendered to a thickness of 40 μm. After vacuum-drying overnight at 60 °C, the resulting electrolyte membrane exhibits a dense and uniform surface morphology (Fig. S29). Silicon stands out as the most promising next-generation anode material due to its natural abundance, high theoretical capacity (e.g., Li_15_Si_4_ at 3572 mAh g^−1^), and moderate operating voltage (approximately 0.4 V vs. Li^+^/Li) [38]. Moreover, silicon-based anodes help mitigate issues such as lithium dendrite formation and short circuits in sulfide-based all-solid-state batteries. In Fig. 5h, i, the all-solid-state NCM83-MPTMS@LPSCBr/μ-Si pouch cell featuring LPSCBr film demonstrates stable cycling with ~92.7% capacity retention after 50 cycles. Galvanostatic charge/discharge curves of the pouch cell with different cycles are shown in Fig. S30, underscoring the practical potential of the modified electrolyte. The MPTMS surface-coating modification strategy for LPSCBr particles provides a reliable route to simultaneously improve the interfacial stability and electrochemical performance of thiophosphate solid electrolytes.

## Conclusions

In summary, a bifunctional MPTMS coating was uniformly deposited on LPSCBr sulfide electrolytes via a scalable mechanochemical ball-milling strategy, realizing excellent moisture resistance and structural stability. The synergistic effect of covalent S–S anchoring and hydrophobic siloxane network enables 93% H_2_S suppression and 83.1% conductivity retention under 30% RH exposure. The MPTMS@LPSCBr electrolyte displays excellent air stability. After 2 h of air exposure, the assembled cells still achieve 84.6% capacity retention over 1000 cycles, far superior to the uncoated sample. Moreover, MPTMS enhances the oxidation stability and interfacial compatibility between the sulfide electrolyte and cathode in composite cathodes. This mechanochemical coating offers a universal, scalable route to improve the environmental stability and electrochemical performance of sulfide solid electrolytes for practical solid-state batteries.

## Supplementary Information

Below is the link to the electronic supplementary material.

Supplementary file1 (DOCX 12249 KB)
