# Supplementary material for: High-Conductivity and Moisture-Tolerant Sulfide Electrolyte Enabled by Bifunctional Coating for Practical All-Solid-State Lithium Batteries
Source: Nanomicro Lett. 2026 Oct 5;19:51. doi: 10.1007/s40820-026-02353-y (PMC13635071; doi:10.1007/s40820-026-02353-y)
Supplement: Supplementary file 1 [file 40820_2026_2353_MOESM1_ESM.docx]

Supporting Information for

**High-Conductivity and Moisture-Tolerant Sulfide Electrolyte Enabled by Bifunctional Coating for Practical All-Solid-State Lithium Batteries**

Dabing Li^1^, Hongjie Xu^2^, Guoqin Cao^1^, Fanfan Liu^1^, Jinjin Ban^1^, Shilin Zhang^1^, Li-Zhen Fan^3,^*** and Junhua Hu^1,^*

^1^ School of Materials Science and Engineering, State Center for International Cooperation on Designer Low-carbon and Environmental Materials (CDLCEM), Zhengzhou University, Zhengzhou 450001, P. R. China

^2^ School of Materials Science and Engineering, North China University of Water Resources and Electric Power, Zhengzhou 450046, P. R. China

^3^ Institute of Advanced Materials and Technology, University of Science and Technology Beijing, Beijing 100083, P. R. China

* Corresponding authors. E-mail: [fanlizhen@ustb.edu.cn](mailto:fanlizhen@ustb.edu.cn) (Li-Zhen Fan); [hujh@zzu.edu.cn](mailto:hujh@zzu.edu.cn) (Junhua Hu)

**S1 Supplementary Text**

***S1.1 Main Materials***

Li_2_S (purity 99.9%) precursor powder was purchased from Alfa Aesar. P_2_S_5_ (purity 99%) precursor powder was obtained from Macklin. LiCl (purity 99.9%) and LiBr (purity 99.9%) precursor powders were purchased from Aladdin. (3-Mercaptopropyl)trimethoxysilane (MPTMS, purity 98%) was acquired from Aladdin and used without further purification for the synthesis of MPTMS@LPSCBr composite. Trimethoxymethylsilane (MTMS) and Butyltrimethoxysilane (BTMS) were both purchased from Macklin and used as received without further purification. All other chemicals were of analytical grade and were used without additional purification. Commercial phase-pure LGPS powder was purchased directly from Shenzhen Kejing Star Technology Company.

***S1.2 Synthesis of Li_5.5_PS_4.5_Cl_0.8_Br_0.7_***

The argyrodite-type Li_5.5_PS_4.5_Cl_0.8_Br_0.7_ was synthesized via a solid-state reaction. Precursor powders of Li_2_S, P_2_S_5_, LiCl, and LiBr, in stoichiometric ratios, were first subjected to high-energy ball milling at 500 rpm for 10 h. The resulting mixture was then sealed under vacuum in a quartz ampoule and underwent a final annealing step at 480 °C for 12 h to crystallize the argyrodite phase.

***S1.3 Synthesis of MPTMS@LPSCBr***

The MPTMS@LPSCBr composite was synthesized using commercially acquired (3-Mercaptopropyl)trimethoxysilane without further purification. The MPTMS@LPSCBr mixture was prepared by planetary mixing of LPSCBr and MPTMS in a 10:1 mass ratio followed by ball milling at 600 rpm for 30 minutes. All material preparation and handling procedures were executed within an argon-filled glovebox maintaining stringent atmospheric controls (H_2_O and O_2_ contents both ≤ 1 ppm). After ball milling, the obtained powder was subjected to vacuum drying at 80 °C for 6 h to remove residual impurities.

***S1.4 Cell assembly and measurements***

All-solid-state lithium batteries (ASSLBs) were fabricated by stacking layers inside a 10 mm diameter polyether ether ketone (PEEK) mold. The composite cathodes were prepared by manually blending cathode active materials (uncoated LiNi_0.83_Co_0.11_Mn_0.06_O_2_, NCM83), lithium-ion conductor (LPSCBr), and vapor grown carbon fiber (VGCF) in an agate mortar for 30 minutes at a mass ratio of 70:30:1. To evaluate the air stability, the assembly process involved first pressing 100 mg of pre-exposed solid electrolyte (LPSCBr or MPTMS@LPSCBr) at 100 MPa to form the electrolyte layer. Subsequently, the as-prepared composite cathode powder was deposited and pressed onto the electrolyte layer at 300 MPa. Finally, a piece of Li_0.5_In alloy (100 μm) was attached as the anode.

For electrochemical performance evaluation, two cathode formulas were applied to distinguish the effect of MPTMS surface modification. The control cathode consisted of NCM83, pristine LPSCBr, and VGCF at a mass ratio of 70:30:1. For the modified cathode, pristine LPSCBr was substituted with pre-synthesized MPTMS@LPSCBr (prepared at a LPSCBr:MPTMS mass ratio of 10:1), while keeping the same NCM83:MPTMS@LPSCBr:VGCF proportion of 70:30:1. For all electrochemical cells, pristine LPSCBr powder (100 mg) was first pressed at 100 MPa to form the base electrolyte layer. The tailored composite cathode powder was subsequently deposited and consolidated at 300 MPa, followed by assembly with a 100 μm-thick Li_0.5_In alloy anode. All cells were tested under a constant stacking pressure of 100 MPa. Galvanostatic charge–discharge measurements were performed on a LAND CT2001A tester within a voltage window of 2.7–4.3 V (vs. Li/Li^+^).

***S1.5 Materials characterization***

The crystal structure of the samples was characterized by X-ray diffraction (XRD, Rigaku D/max-RB) using Cu-Kα radiation (λ = 1.54178 Å) at room temperature. Data were acquired in the 2θ range of 10°–90° with a step size of 0.02° and a counting time of 0.5 s per step, followed by Rietveld refinement using the GSAS II software. To prevent degradation, the samples were shielded with a polyimide film during handling. Fourier Transform Infrared (FTIR) spectroscopy was performed on a Thermo Scientific Nicolet 6700 spectrometer, covering the wavenumber range of 400 to 4000 cm^−1^. Raman scattering measurement was conducted on a JY HR800 instrument using a 532 nm laser as the excitation source. Morphology and elemental distribution were analyzed by field-emission scanning electron microscopy (FE-SEM, JEOL JSM-7001F) coupled with energy-dispersive spectroscopy (EDS). Chemical states were probed by X-ray photoelectron spectroscopy (XPS) on a Thermo Fisher ESCALAB 250Xi instrument with a monochromatic Al Kα source; all binding energies were referenced to the adventitious C 1s peak at 284.8 eV. The microstructure was further examined by transmission electron microscopy (TEM, JEOL 3200FS), noting that the specimens were exposed to ambient air for less than 60 seconds during transfer. Solid-state NMR measurements were conducted on a Bruker Avance III 800 MHz (18.8 T) spectrometer. All samples were handled in an argon atmosphere to prevent moisture exposure. Thermogravimetric analysis (TGA) was carried out using a NETZSCH STA 449 F3 synchronous thermal analyzer with dry N_2_ flow, at a heating rate of 10 °C min^−1^ from 30 to 800 °C.

***S1.6 Electrochemical measurements***

Electrochemical impedance spectroscopy (EIS) was measured using a Solartron 1260+1287 station with a 10 mV amplitude over a frequency range of 0.1 Hz to 1 MHz. The electronic conductivity was determined via direct current polarization using a stainless steel/SE/stainless steel symmetrical cell under 0.4 V. The electrochemical stability of LPSCBr and MPTMS@LPSCBr solid electrolytes was assessed by cyclic voltammetry (CV), using a CHI660B workstation at a scan rate of 0.2 mV s^−1^ between 2 and 5.0 V (vs. Li⁺/Li). First, a dense electrolyte layer was prepared by pressing 100 mg LPSCBr powder in a 10 mm PEEK mold at 300 MPa. Then, the working electrode consisted of 10 mg of a mixture of the respective electrolyte and carbon black (7:3 by mass), paired with a Li metal counter/reference electrode.

***S1.7 Air-stability measurements***

The air stability of the solid electrolyte was assessed by quantifying the H_2_S gas released under controlled humid atmospheres, including ambient air and oxygen-free N_2_ carrier gas with fixed relative humidity. The samples were exposed to two distinct humid environments at room temperature for parallel comparison: (1) natural ambient air with 30% RH; (2) pure oxygen-free N_2_ gas tuned to 30% RH to isolate the sole effect of moisture without O_2_ interference. Precise sample masses (100 mg of bare LPSCBr or MPTMS@LPSCBr) were placed separately in identical sealed vessels fitted with an H_2_S detector (BH-90A, Baoshian) and an internal stirring fan to guarantee uniform gas distribution inside the chamber. Each vessel was maintained at room temperature for designated aging durations ranging from 2 h to 10 h, during which the internal relative humidity was recorded continuously in real time. The instantaneous H_2_S concentration released from the electrolyte powders was directly acquired via the H_2_S sensor, and cumulative H_2_S emission was calculated for quantitative comparison between air-aged and N_2_-humidified groups. All experimental parameters including sample mass, chamber volume, gas flow rate and holding temperature were strictly identical for the two humid environments to guarantee reliable parallel comparison. After scheduled aging treatments, powders were collected inside an Ar-filled glovebox for subsequent XRD characterization to identify hydrolysis and oxidation-derived degradation products.

***S1.8 Preparation of MPTMS@LPSCBr film***

Polyisobutylene (PIB, average molecular weight 800,000) was purchased from Aladdin and adopted as the binder for sulfide electrolyte films. The PIB powder was dissolved in anhydrous toluene (99.9%, Aladdin) to yield a 5 wt% binder solution.

Subsequently, pre-synthesized MPTMS@LPSCBr powder and PIB adhesive solution were mechanically ball-milled at 600 rpm for 30 min to form a homogeneous slurry. The MPTMS@LPSCBr powder was pre-fabricated with a fixed LPSCBr:MPTMS mass ratio of 10:1 before slurry preparation. The as-obtained slurry was coated onto a release polyethylene terephthalate (PET) substrate via the doctor-blade method. The thickness of the green sulfide film was tuned to ~40 μm by calendering, followed by overnight vacuum drying at 60 °C. Before electrochemical characterization, the electrolyte film was cold-pressed at 100 MPa for 5 min and punched into circular discs with a diameter of 10 mm. All film fabrication procedures were carried out inside an Ar-filled glove box with H_2_O and O_2_ levels maintained below 1 ppm.

***S1.9 All-solid-state lithium pouch cells***

The NCM83 composite cathode was fabricated via a solvent-free route. Pre-synthesized MPTMS@LPSCBr powder (LPSCBr:MPTMS mass ratio = 10:1) was mixed with NCM83 and VGCF at a mass ratio of 70:30:1, followed by blending with 0.5 wt% polytetrafluoroethylene (PTFE) binder. The mixture was rolled into a thin cathode foil with an areal mass loading of 8.92 mg cm^−2^. For micron-sized silicon anodes, a homogeneous slurry was prepared by dispersing micro-Si particles, carbon black, and polyvinylidene fluoride (PVDF) binder at a mass ratio of 98:1:1 in N-methyl-2-pyrrolidone (NMP). The obtained slurry was cast onto a copper current collector via blade coating, then vacuum-dried at 80 °C overnight to fully remove residual solvent. Subsequently, the as-fabricated NCM83 cathode foil, freestanding MPTMS@LPSCBr electrolyte film, and μ-Si anode foil were stacked and hot-pressed sequentially to assemble an all-solid-state pouch cell with an N/P ratio of 1.2. The laminated cell was vacuum-sealed inside an aluminum plastic composite pouch. Galvanostatic charge–discharge measurements were carried out on a LAND battery tester (CT2001A) within a voltage window of 2.6–4.25 V (vs. Li/Li^+^). All electrode and pouch cell assembly procedures were conducted in an argon-filled glove box where H_2_O and O_2_ contents were maintained below 1 ppm.

***S1.10 Theoretical Calculations***

Theoretical computations were conducted using the Vienna Ab Initio Simulation Package (VASP) [S11, S12]. The electron-ion interaction was described by the projector-augmented-wave (PAW) approach [S13]. The exchange-correlation functional was treated with the Perdew−Burke−Ernzerhof (PBE) functional generalized gradient approximation (GGA) [S14]. Long-range van der Waals interactions were incorporated using the DFT-D3 correction method [S15, S

16]. To model the interaction between the LPSCBr surface and organic molecules (MPTMS and MTMS), a surface slab model with a vacuum layer of 15 Å was constructed to prevent spurious interactions between periodic images. A plane-wave kinetic energy cutoff of 450 eV was adopted. All atomic positions were fully relaxed until the total energy and atomic forces converged to 1 × 10^−5^ eV and 0.03 eV/Å, respectively. A Monkhorst–Pack grid with a 3×3×1 k-point mesh was used to sample the first Brillouin zone.

**S2 Supporting Figures**

**Fig. S1** The X-ray diffraction pattern of LPSCBr and the corresponding Rietveld refinements.

**Fig. S2** ^31^P spin echo solid-state NMR spectra collected on MPTMS@LPSCBr.

**Fig. S3** SEM and EDS mapping images of MPTMS@LPSCBr powders.

**Fig. S4** Surface SEM morphologies of cold-pressed pellets: **a** pristine LPSCBr, **b** MPTMS@LPSCBr.

**Fig. S5 a** Surface SEM image of cold-pressed MPTMS@LPSCBr electrolyte pellet; **b-d** EDS elemental distribution maps of P, S and Si, respectively.

**Fig. S6** Thermogravimetric analysis of LPSCBr and MPTMS@LPSCBr powders.

**Fig. S7** TGA curves of MPTMS-modified LPSCBr composite electrolytes with different mass ratios: **a** LPSCBr:MPTMS = 20:1, **b** LPSCBr:MPTMS = 5:1.

**Fig. S8** Arrhenius plots of LPSCBr powders.

**Fig. S9** Nyquist plots of pristine LPSCBr and MPTMS-coated LPSCBr at different mass ratios.

**Fig. S10** ^7^Li spin echo solid-state NMR spectra collected on LPSCBr and MPTMS@LPSCBr.

**Fig. S11** Time-dependent cumulative H_2_S release curves of MPTMS@LPSCBr at different mass ratios.

**Fig. S12** Optimized atomic structures of the four systems of interest in this work. **a** Li_5.5_PS_4.5_Cl_0.8_Br_0.7_ (LPSCBr), **b** Trimethylmethoxysilane (MTMS), **c** Butyltrimethoxysilane (BTMS), and **d** (3-Mercaptopropyl)trimethoxysilane (MPTMS). The nine atomic types are represented by colored spheres as follows: O (red), Si (blue), C (gray), H (white), P (bright purple), S (yellow), Br (pink), Cl (green), Li (cyan).

**Fig. S13** Time amount changes in H_2_S gas curves for BTMS- and MTMS-coated LPSCBr samples (LPSCBr:hydrophobic molecules = 10:1 mass ratio)

**Fig. S14** Time-dependent cumulative H_2_S release volume of pristine Li_10_GeP_2_S_12_ and MPTMS-modified Li_10_GeP_2_S_12_ (mass ratio = 10:1) under ambient humid air exposure.

**Fig. S15** XRD spectrum of bare LPSCBr exposed to 30% RH N_2_ atmosphere for 2 h.

**Fig. S16** XRD spectrum of bare LPSCBr exposed to 30% RH N_2_ atmosphere for 10 h.

**Fig. S17** The charge/discharge curves of NCM83/Li-In cell using exposed LPSCBr electrolytes at different current densities.

**Fig. S18** The charge/discharge curves of NCM83/Li-In cell using exposed MPTMS@LPSCBr electrolytes at different current densities.

**Fig. S19** The trend of interfacial impedance over 50 cycles for cell assembled after exposing LPSCBr to air at 30% relative humidity for 2 h.

**Fig. S20** CV profiles of the LPSCBr/C composite electrodes at a scan rate of 0.2 mV s^−1^.

**Fig. S21** CV profiles of the MPTMS@LPSCBr/C composite electrodes at a scan rate of 0.2 mV s^−1^.

**Fig. S22** Electronic conductivity of pristine LPSCBr and MPTMS-modified LPSCBr with different LPSCBr:MPTMS mass ratios.

**Fig. S23** **a** Galvanostatic charge/discharge curves of the NCM83-MPTMS@LPSCBr/LPSCBr/Li-In and **b** NCM83-LPSCBr/LPSCBr/Li-In cell with an areal mass loading of 8.92 mg cm^−2^ and 0.1 C with different cycles.

**Fig. S24** DRT analysis of NCM83-LPSCBr/LPSCBr/Li-In cell during **a** charging and **b** discharging at the first cycle.

**Fig. S25** DRT analysis of NCM83-MPTMS@LPSCBr/LPSCBr/Li-In cell during **a** charging and **b** discharging at the first cycle.

**Fig. S26 a** S 2p and **b** P 2p XPS spectra of the bare LPSCBr/NCM83 electrode stack before and after 50 cycles.

**Fig. S27** XPS spectra of the MPTMS@LPSCBr/NCM83 electrode stack before and after 1000 cycles: **a** P 2p, **b** S 2p, **c** Si 2p.

**Fig. S28** Galvanostatic charge/discharge curves of the NCM83-MPTMS@LPSCBr/LPSCBr/Li-In cell with an areal mass loading of 26.75 mg cm^−2^ and 0.1 C with different cycles.

**Fig. S29 a** SEM image, **b** cross-sectional SEM image of the MPTMS@LPSCBr film.

**Fig. S30** Galvanostatic charge/discharge curves of the all-solid-state NCM83-MPTMS@LPSCBr/LPSCBr film/μ-Si pouch cell at 0.1 C with different cycles.

**S3 Supplementary Tables**

**Table S1** Summary of literature data on the air stability of sulfide electrolytes

| Sulfide SEs | Coating materials | Thickness/nm | Li^+^ conductivity retention | Air exposure condition | Ref. |
| --- | --- | --- | --- | --- | --- |
| Li_9.98_Ge_0.99_Sn_0.01_P_2_S_11.98_F_0.02_ | - | - | 35.2% | RH 35-40%, 20min | [S1] |
| Li_6.04_P_0.98_Bi_0.02_S_4.97_O_0.03_Cl | - | - | 26.8% | RH 20%, 60 min | [S2] |
| Li_6.16_P_0.92_In_0.08_S_4.88_O_0.12_Cl | - | - | 86.9% | RH 20%, 60 min | [S3] |
| Li_5.6_Cu_0.2_PS_4.8_Br_1.2_ | - | - | 85.2% | RH 30%, 60 min | [S4] |
| Li_6_P_0.95_Sb_0.05_S_4.875_O_0.125_Cl | - | - | 78% | RH 35%, 30 min | [S5] |
| Li_10_GeP_2_S_12_ | LiF | ~10 | 57.5% | RH 30%, 40 min | [S6] |
| Li_7_P_2_S_8_Br_0.5_I_0.5_ | 1-bromopentane | 10 | 89.2% | RH 0.5%, 120min | [S7] |
| Li_6_PS_5_Cl | PEGDME-LiTFSI | 5-10 | 54.2% | RH 3%, 10 h | [S8] |
| Li_6_PS_5_Cl | F-PDMS | ~ 5 | 49% | RH 0.2%, 72 h | [S9] |
| Li_6_PS_5_Cl | CO_2_ | 10-40 | 20.8% | RH 17%, 1 h | [S10] |
| Li_5.5_PS_4.5_Cl_0.8_Br_0.7_ | MPTMS | ~10 | 52.4% | RH 30%, 600 min | This work |

**Table S2** Room-temperature ionic conductivity and 35-min H_2_S release amount of the MPTMS-modified LPSCBr electrolyte from three independent batches.

| Batch | Room-temperature ionic conductivity (mS·cm^−1^) | H_2_S release amount in 35 min (cm^3^·g^−1^) |
| --- | --- | --- |
| 1 | 10.72 | 0.32 |
| 2 | 10.85 | 0.34 |
| 3 | 10.79 | 0.33 |
| Average | 10.79±0.07 | 0.33±0.01 |

The relative deviations of all three metrics are below 2%, which verifies that the mechanochemical coating process features excellent batch-to-batch reproducibility and holds promising potential for large-scale production.

**Supplementary References**

[S1] N. Zhang, Q. He, L. Zhang, J. Zhang, L. Huang et al. Homogeneous fluorine doping toward highly conductive and stable Li_10_GeP_2_S_12_ solid electrolyte for all‐solid‐state lithium batteries. Adv. Mater. **36**, 2408903 (2024). <https://doi.org/10.1002/adma.202408903>
[S2] H. Liu, Q. Zhu, C. Wang, G. Wang, Y. Liang et al. High air stability and excellent Li metal compatibility of argyrodite-based electrolyte enabling superior all-solid-state Li metal batteries. Adv. Funct. Mater. **32**, 2203858 (2022). <https://doi.org/10.1002/adfm.202203858>
[S3] C. Wang, J. Hao, J. Wu, H. Shi, L. Fan et al. Enhanced air stability and Li metal compatibility of Li-argyrodite electrolytes triggered by In_2_O_3_ Co-doping for all-solid-state Li metal batteries. Adv. Funct. Mater. **34**, 2313308 (2024). <https://doi.org/10.1002/adfm.202313308>
[S4] Z. Jiang, Y. Liu, H. Peng, J. Li, X. Xu et al. Enhanced air stability and interfacial compatibility of Li-argyrodite sulfide electrolyte triggered by CuBr co-substitution for all-solid-state lithium batteries. Energy Storage Mater. **56**, 300-309 (2023). <https://doi.org/10.1016/j.ensm.2023.01.018>
[S5] C. Liu, T. Zhang, R. Wang, B. Chen, D. Wang et al. Regulating p-band center of sulfur in Li-argyrodite to stabilize dual solid-solid interface for robust all-solid-state lithium–sulfur battery. Adv. Funct. Mater. **35**, 2412144 (2025). https://doi.org/10.1002/adfm.202412144
[S6] Y. Jin, Q. He, G. Liu, Z. Gu, M. Wu et al. Fluorinated Li_10_GeP_2_S_12_ enables stable all-solid-state lithium batteries. Adv. Mater. **35**, 2211047 (2023). <https://doi.org/10.1002/adma.202211047>
[S7] Z. Yu, S.-L. Shang, K. Ahn, D. T. Marty, R. Feng et al. Enhancing moisture stability of sulfide solid-state electrolytes by reversible amphipathic molecular coating. ACS Appl. Mater. Interfaces **14**, 32035-32042 (2022). <https://doi.org/10.1021/acsami.2c07388>
[S8] X. Yang, X. Gao, M. Jiang, J. Luo, J. Yan et al. Grain boundary electronic insulation for high-performance all-solid-state lithium batteries. Angew. Chem. Int. Ed. **62**, e202215680 (2023). <https://doi.org/10.1002/anie.202215680>
[S9] K. T. Kim, J. Woo, Y.-S. Kim, S. Sung, C. Park et al. Ultrathin superhydrophobic coatings for air-stable inorganic solid electrolytes: toward dry room application for all-solid-state batteries. Adv. Energy Mater. **13**, 2301600 (2023). <https://doi.org/10.1002/aenm.202301600>
[S10] X. Zhang, X. Li, S. Weng, S. Wu, Q. Liu et al. Spontaneous gas-solid reaction on sulfide electrolytes for high-performance all-solid-state batteries. Energy Environ. Sci. **16**, 1091-1099 (2023). <https://doi.org/10.1039/D2EE03358E>
[S11] G. G. Kresse, J. J. Furthmüller. Efficient iterative schemes for Ab initio total-energy calculations using a plane-wave basis set. Physical review. B, Condensed matter **54**, 11169 (1996). <https://doi.org/10.1103/physrevb.54.11169>
[S12] G. Kresse, D. Joubert. From ultrasoft pseudopotentials to the projector augmented-wave method. Phys. Rev. B **59**, 1758-1775 (1999). <https://doi.org/10.1103/PhysRevB.59.1758>
[S13] P. E. Blochl. Projector augmented-wave method. Phys. Rev. B Condens. Matter **50**, 17953-17979 (1994). <https://doi.org/10.1103/PhysRevB.50.17953>
[S14] John, P., Perdew, Kieron, Burke et al. Generalized gradient approximation made simple. Phys. Rev. Lett. **78**, 1396 (1996). <https://doi.org/10.1103/physrevlett.77.3865>
[S15] S. Grimme, J. Antony, S. Ehrlich & H. Krieg. A consistent and accurate ab initio parametrization of density functional dispersion correction (DFT-D) for the 94 elements H-Pu. J. Chem. Phys. **132**, 154104 (2010). <https://doi.org/10.1063/1.3382344>
[S16] S. Grimme, S. Ehrlich & L. Goerigk. Effect of the damping function in dispersion corrected density functional theory. J. Comput. Chem. **32**, 1456-1465 (2011). <https://doi.org/10.1002/jcc.21759>
